# An Unbiased Geometry‐Resolved Membrane Platform for Proteome‐Scale Discovery of Membrane Curvature Sensors

**DOI:** 10.1002/advs.77654

**Published:** 2026-09-08

**Authors:** Takumi Komikawa, Rikuto Kawakami, Kunanon Chattrairat, Tatsuya Niwa, Taiga Ajiri, Takao Yasui, Masayoshi Tanaka

**Affiliations:** ^1^ School of Materials and Chemical Technology Institute of Science Tokyo Yokohama Japan; ^2^ Graduate School of Engineering Nagoya University Nagoya Japan; ^3^ Institute of Integrated Research Institute of Science Tokyo Yokohama Japan

**Keywords:** geometry‐resolved platform, membrane curvature sensor, proteome analysis, supported lipid bilayer

## Abstract

Membrane curvature organizes protein‐driven biochemistry on cellular membranes. Conventional membrane curvature assays are inherently limited by both candidate‐based experimental designs and restricted membrane geometries, preventing proteome‐scale discovery of curvature‐sensing proteins. Here, we present a geometry‐resolved membrane platform that enables hypothesis‐agnostic identification of curvature‐sensing proteins. A supported lipid bilayer on a hemispherical microwell array simultaneously presents convex rims, concave well interiors, and flat planar regions, while providing sufficient membrane surface area for shotgun proteomics. Following validation using canonical BAR domain proteins, we applied the platform to proteomic screening of a cell‐derived peripheral membrane protein fraction. This analysis identified 671 proteins, including 71 curvature‐responsive candidates spanning preferences for positively curved, negatively curved, and flat membranes. Subsequent candidate prioritization using a protein–protein interaction‐guided workflow, followed by validation with purified proteins, demonstrated that the platform provides a comprehensive pipeline linking proteomics‐based discovery to imaging‐based evaluation. By interrogating membranes of positive, negative, and zero curvature in parallel, this platform eliminates geometric bias, expands the experimentally accessible landscape of membrane curvature sensing, and enables the discovery of previously overlooked modes of membrane curvature recognition.

## Graphical abstract

A novel geometry‐resolved membrane platform provides a seamlessly integrated pipeline for studying curvature‐sensing proteins. By linking shotgun proteomics with quantitative imaging, this approach enables both the unbiased discovery and rigorous validation of novel sensors across the entire curvature range—positive, negative, and zero.

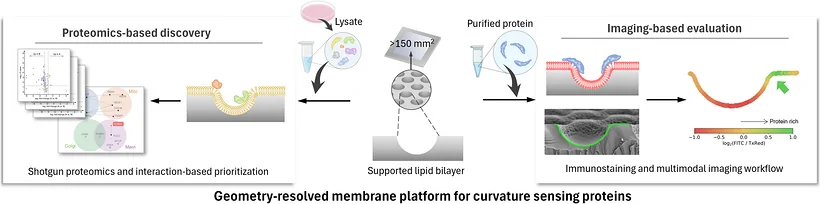

## 1 Introduction

Cellular membranes do not simply partition cellular contents; their local geometry actively organizes the protein‐driven biochemistry that proceeds on them. During endocytosis, autophagy, and organelle biogenesis, membranes are continuously reshaped across geometries ranging from highly curved tubules and vesicles to extended planar sheets [1, 2, 3, 4]. Understanding how proteins recognize these diverse membrane geometries has therefore become an essential question in cell biology [5]. Curvature sensing—the preferential association of proteins according to membrane curvature—is best exemplified by amphipathic helices such as ALPS (Amphipathic Lipid Packing Sensor) motifs [6] or the BAR (Bin/Amphiphysin/Rvs) domain superfamily [7, 8], both of which recognize curved surfaces. It is conventionally assessed in vitro using membranes of artificially defined geometry such as liposomes [7, 9], nanotubes [10, 11], and supported lipid bilayers [12, 13, 14]. Yet these systems share two important limitations. More fundamentally, each method presents only a single sign and a narrow range of curvature and rarely allows direct comparison with a flat membrane, so proteins preferring geometries outside the tested range remain undetectable. In addition, they require candidate proteins to be purified or labeled in advance, making the assays hypothesis‐driven and low‐throughput: only proteins already suspected of sensing curvature are ever tested. As a result, unbiased proteome‐scale discovery across the landscape of membrane curvature sensors has remained out of reach.

Overcoming both biases at once requires transforming curvature‐sensing assays from candidate‐based approaches into a proteome‐scale discovery platform. Proximity‐labeling proteomics such as BioID [15], APEX [16], and TurboID [17] achieved a similar conceptual advance by converting spatial proximity associated with a defined biological process into an unbiased, proteome‐wide readout. Instead of tagging proteins near a bait protein, a curvature‐based screen exploits differential affinity for defined membrane geometries to directly capture the curvature‐responsive proteins from a complex lysate. Although a previous proteome‐scale study identified BAR‐domain arfaptins using clathrin/AP‐1‐coated tubular liposomes generated by actin polymerization [18], membrane curvature itself was not an independently controlled experimental variable. To isolate curvature, an engineered substrate that decouples geometry from membrane chemistry is required, enabling systematic comparison across positively curved, negatively curved, and flat membranes under a uniform lipid composition. We previously implemented this concept on silica‐bead‐supported lipid bilayers [19], but such spherical models provide only positively curved (convex) membranes. Extending proteome‐scale discovery across positive, negative, and zero curvature therefore requires a purpose‐engineered solid substrate.

Existing nanostructured substrates overcome some of the geometric limitations, but remain fundamentally constrained by membrane area and imaging‐based readout. Platforms based on nanopillars [20, 21], nanobars [22], or engineered concave features such as NanoCurvS [23] impose defined curvatures on a supported bilayer and read out protein enrichment by fluorescence microscopy. In these designs, curved membrane regions are largely confined to the vertical sidewalls, leaving most of the membrane flat. Increasing the curved membrane area therefore requires densely packed, high‐aspect‐ratio features, which in turn hinder the formation of a continuous supported bilayer. More fundamentally, imaging‐based assays sample only a few hundred square micrometers—sufficient to characterize a single protein, but far below the membrane area needed to recover enough material for mass spectrometry. Bridging single‐protein curvature assays to proteome‐scale identification therefore calls for a substrate that presents positive, negative, and zero curvature simultaneously on a single continuous bilayer, while scaling to the areas required for shotgun proteomics.

Here we present a geometry‐resolved membrane platform that meets these requirements, based on supported lipid bilayers formed on hemispherical microwell arrays. A single continuous bilayer presents convex (rims), concave (well interiors), and flat (inter‐well) regions side by side. Since the three curvatures coexist within a single field of view, curvature preference can be read out via fluorescence microscopy relative to an internal lipid fluorescence reference. Furthermore, because curvature is distributed across the entire well surface rather than confined to vertical sidewalls, a substantial proportion of curved membrane area is achieved at a low aspect ratio, thereby preserving bilayer continuity and facilitating easy scale‐up to the area required for mass spectrometry‐based assays. We benchmarked the platform with canonical BAR‐domain sensors before applying it to proteome‐scale screening of cellular peripheral membrane proteins, followed by validation of prioritized candidates using purified proteins. Because the workflow requires no preselection and scores every protein on a membrane displaying positive, negative, and zero curvatures, it revealed both a conventional positive‐curvature sensor and a previously overlooked class. These proteins preferentially associate with curvature regimes that lie outside the window interrogated by established assays. A scalable, geometry‐resolved membrane platform thus expands the experimentally accessible landscape of membrane curvature sensing and provides a versatile strategy for investigating cellular machineries organized by membrane geometry.

## 2 Results

### 2.1 Platform Design for Geometry‐Resolved Proteomic Screening

To enable geometry‐resolved proteome‐scale discovery, the substrate must satisfy three design requirements. Because membrane‐associated proteins are recovered from the substrate in bulk for mass spectrometry rather than from defined locations, proteomic comparison must be performed between substrates with systematically varied membrane geometries. The substrate should (i) provide sufficient curved membrane area for proteomics, (ii) independently tune convex, concave, and flat membrane fractions, and (iii) support a continuous bilayer scalable for proteomics.

Existing nanostructured curvature platforms cannot satisfy some of these requirements (Table 1). First, increasing the curved fraction requires high‐aspect‐ratio structures that may hinder uniform bilayer coating. Second, concave features designed for negative curvature inevitably generate positive curvature at their rims, inherently coupling the negative and positive areas.

**TABLE 1:** Substrate strategies for presenting defined membrane curvature to a supported lipid bilayer.

Platform	Curvature‐defining plane	Curvature signs	(i) Dominant curved area	(ii) Neg/Pos ratio tunable	(iii) Area scalable for MS	Refs.
Spherical SLB (beads)	— (beads surface)	Pos	Yes	—	Yes	[19]
Nanopillar	*XY* (sidewall)	Pos^1^	No	—	No	[20]
Nanobar	*XY* (sidewall)	Pos + Zero	No	—	No	[22]
NanoCurvS (concave “U”)	*XY* (sidewall)	Pos + Neg + Zero	No	No^2^	No	[23]
Wavy substrate	*XZ* (topography)	Pos + Neg	Yes	No (fixed ratio)	Yes	[14]
This work (hemispherical microwell)	*XZ* (topography)	Pos + Neg + Zero	Yes (tunable via pitch)	Yes	Yes	

^1^ Flat region dominates but serves as background, not a controlled coexisting reference in one field.

^2^ Negative curvature is accompanied by positive curvature at outer rims, precluding independent area control.

Our topography‐based design satisfies all three requirements (Table 1). In this design, on a hemispherical microwell array, curvature is distributed across the entire well surface, allowing a substantial proportion of curved membrane area and a uniform lipid bilayer to be achieved over areas exceeding 150 mm^2^—the scale required for mass spectrometry (Supplementary Note). The geometry is set by two independently controlled parameters: the well radius, which fixes curvature magnitude, and the well pitch, which sets the curved‐area ratio and the relative abundance of each geometry. Varying these parameters yields the graded curvature‐density substrate series used for comparative proteomics. These design principles formed the basis of the geometry‐resolved membrane platform and the discovery workflow evaluated throughout this study.

**FIGURE 1:**
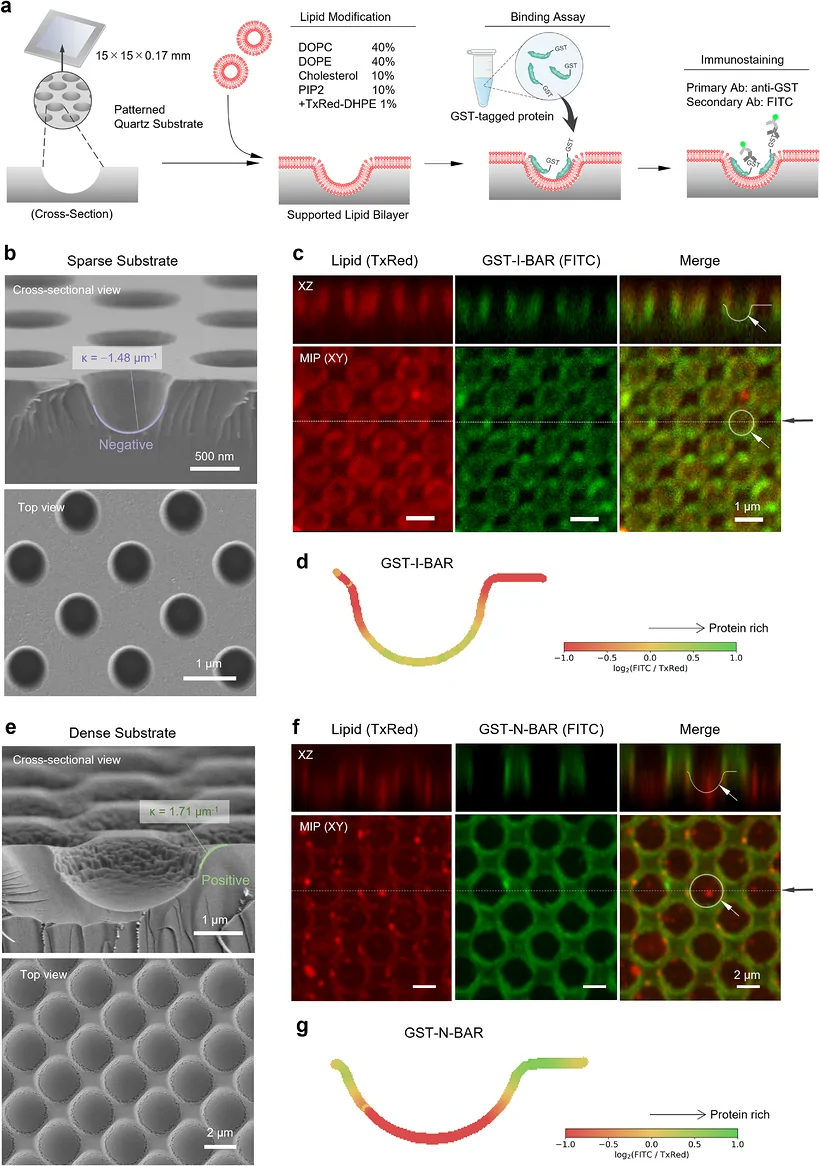
In vitro curvature‐sensing evaluation of I‐BAR (IRSp53) and N‐BAR (Amphiphysin) domains on patterned supported lipid bilayers. (a) Schematic illustration of the experimental workflow: fabrication of a supported lipid bilayer (SLB) on a nanopatterned quartz substrate, followed by a binding assay with GST‐tagged BAR‐domain proteins and immunofluorescence staining. (b,e) Representative SEM images of the sparse (b) and dense (e) quartz substrates. Upper panels: tilted cross‐sectional views; lower panels: top views. Fitted curvature values at the well bottom or the well edge (negative, purple in (b); positive, green in (e)) are indicated. Scale bars are shown in each panel. (c,f) Representative confocal fluorescence images of protein binding on sparse (c) and dense (f) substrates. Top rows: X–Z orthogonal views. Bottom rows: maximum intensity projections (MIP, XY). Red and green channels represent lipids (Texas Red–DHPE) and proteins (FITC‐conjugated secondary antibody), respectively. Black arrows indicate the positions of the XZ cross‐sections; white arrows in the merged images indicate well positions. Scale bars, 1 µm (c) and 2 µm (f). (d,g) Spatial distribution of protein enrichment, defined as log_2_(I_FITC_/I_TexasRed_), mapped onto the substrate surface topography. Profiles were derived from spatially averaged images of *n* = 30 individual patterned wells for GST–I‐BAR (d) and GST–N‐BAR (g).

**FIGURE 2:**
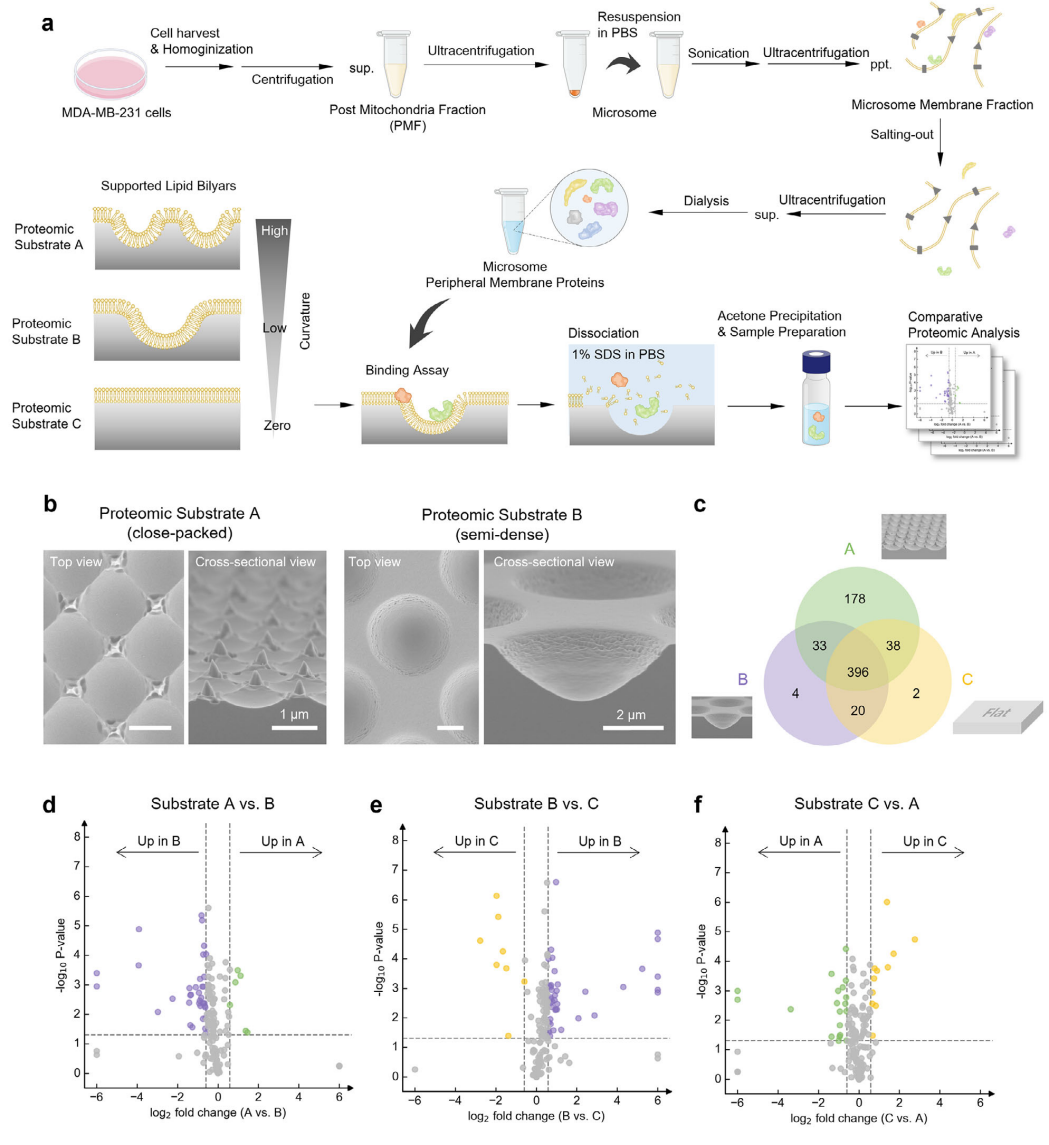
Proteomic screening for curvature‐sensing peripheral membrane proteins using patterned SLBs. (a) Schematic workflow of the experimental procedure, including the extraction of the microsome membrane protein fraction from MDA‐MB‐231 cells and the subsequent binding assay on curvature‐patterned SLBs. (b) Representative SEM images of quartz substrates with Substrate A (close‐packed; left) and B (semi‐dense; right). For each substrate, top views and tilted cross‐sectional views are shown. Scale bars: 1 µm (left) and 2 µm (right). (c) Venn diagram of proteins identified by mass spectrometry across the three SLB geometries (A–C). Substrate C is a flat (zero‐curvature) control. (d–f) Volcano plots comparing protein abundance between SLB geometric pairs: A vs. B (d), B vs. C (e), and C vs. A (f). Colored data points represent proteins with significant differential enrichment (two‐sided Student's *t*‐test with Benjamini–Hochberg FDR correction, adjusted *p*< 0.05 and |fold change| >1.5; n = 3 technical replicates from a single biological preparation).

**FIGURE 3:**
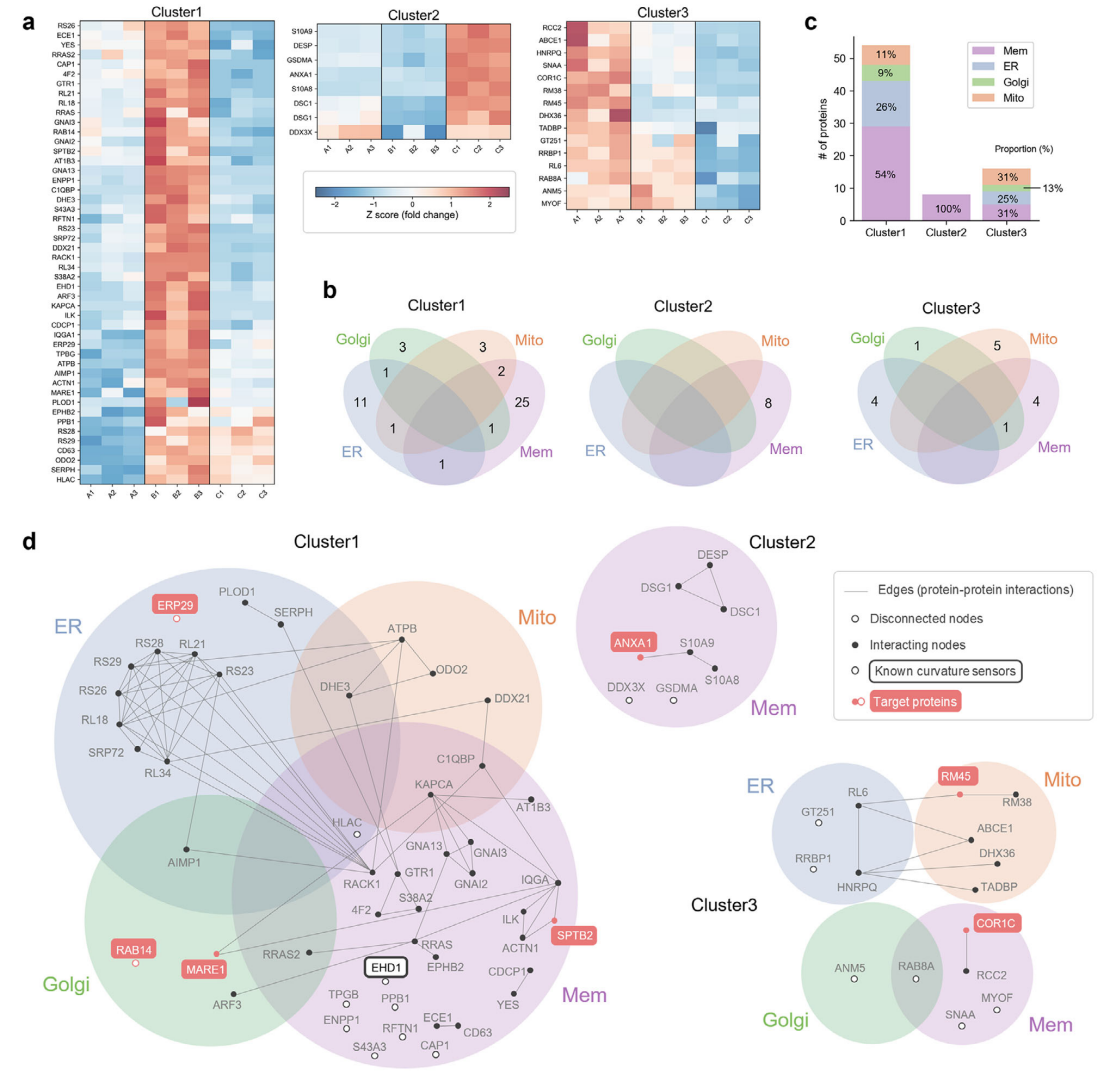
Bioinformatic prioritization of curvature‐sensing protein candidates. (a) Hierarchical clustering (k = 3 clusters) based on the normalized abundance of identified proteins across different substrate geometries; x‐axis: substrate type and technical replicates (e.g., A1–A3 represent three independent mass spectrometry runs from a single biological sample on substrate A). Data are presented as *Z*‐score‐normalized abundance. (b,c) Subcellular localization of proteins within each cluster. (b) Venn diagram showing the number of proteins assigned to different localization categories. (c) Proportion of proteins in each category. Proteins assigned to multiple categories are counted in each applicable category. (d) Protein–protein interaction (PPI) networks for each cluster based on the STRING database. Black circles represent interacting nodes (proteins with at least one interaction), while white circles represent disconnected nodes. Target proteins selected for subsequent analysis are outlined in red.

**FIGURE 4:**
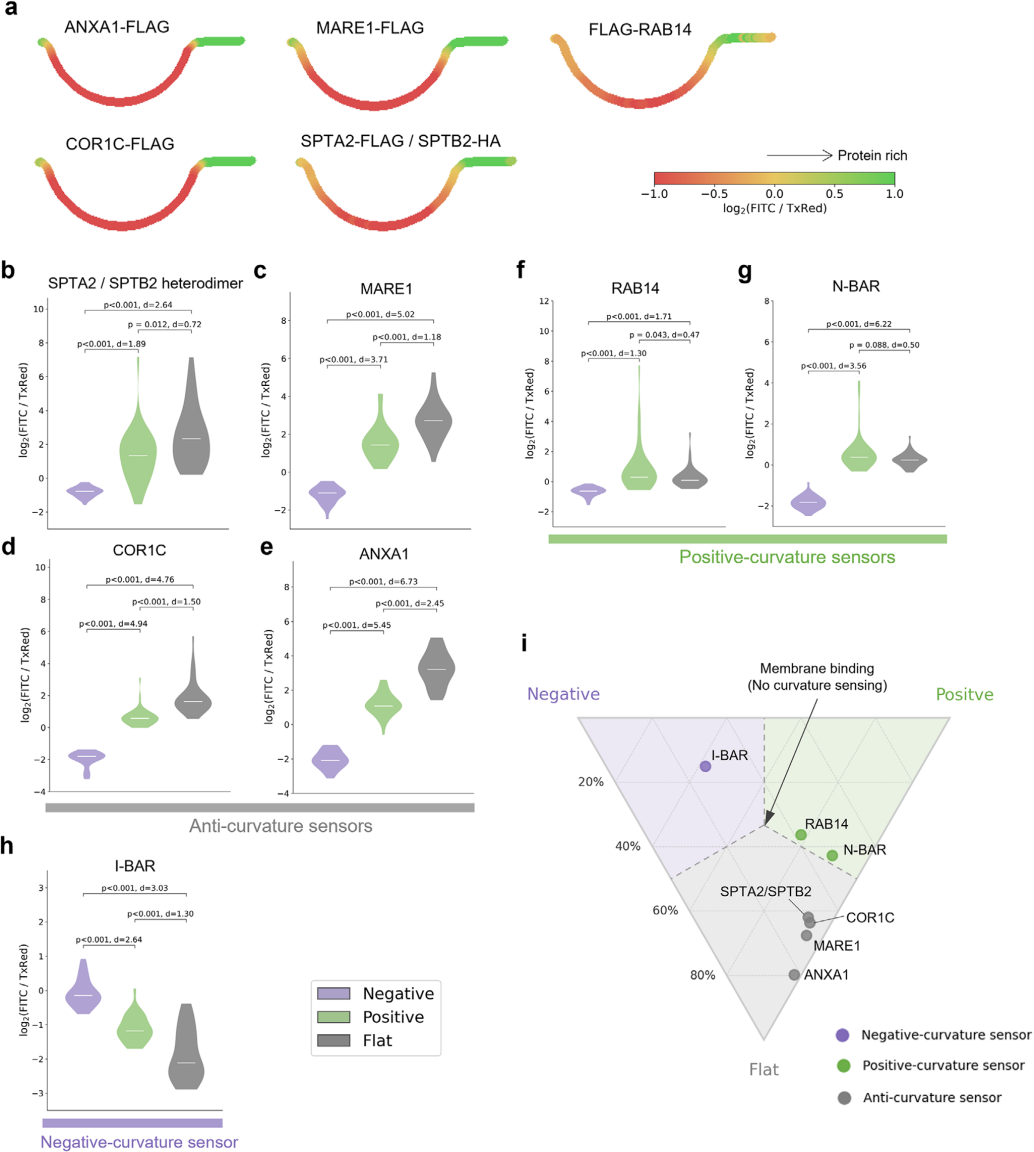
In vitro validation of curvature‐sensing properties of candidate proteins identified by proteomic screening. (a) Spatial distribution of the protein enrichment factor, defined as log_2_(I_FITC_/I_TexasRed_), mapped onto the substrate surface topography. Profiles are derived from spatially averaged images of *n* ≥30 individual patterned wells per protein. (b–h) Quantitative comparison of protein enrichment across negative‐curvature, positive‐curvature, and flat regions. Data are presented as violin plots with horizontal lines indicating medians (*n* ≥30 wells per condition). Statistical significance was assessed using two‐sided Mann–Whitney U tests; effect sizes are reported as Cohen's *d*. Proteins are classified as anti‐curvature sensors (SPTA2/SPTB2 heterodimer, MARE1, COR1C, and ANXA1; b–e), positive‐curvature sensors (RAB14 and N‐BAR; f,g), or a negative‐curvature sensor (I‐BAR; h). (i) Ternary plot summarizing the curvature‐sensing classification. Raw enrichment values for flat, negative and positive regions were converted to proportional weights using a base‐2 softmax transformation ($w_{i}=2^{x_{i}}/\sum_{j}2^{x_{j}})$, preserving the ratio relationships inherent in log‐scale data. Each protein is plotted within an inverted triangle whose vertices correspond to flat (bottom), negative (upper left), and positive (upper right). Dashed lines connecting the centroid to the midpoints of each edge divide the triangle into three classification regions; the centroid represents membrane binding without curvature preference. Purple circles: negative‐curvature sensors; green circles: positive‐curvature sensors; grey circles: anti‐curvature sensors.

The platform was validated through a workflow comprising benchmarking with canonical BAR‐domain sensors (Figure 1), proteome‐wide screening across the graded curvature‐density substrate series (Figure 2), prioritization of direct curvature‐sensing candidates (Figure 3), and validation using purified proteins (Figure 4).

### 2.2 Fabrication of the Supported Lipid Bilayers and Benchmarking Against Canonical Curvature Sensors

To establish a platform suitable for proteome‐scale screening, we fabricated two hemispherical microwell arrays on quartz by grayscale photolithography and reactive‐ion etching, and characterized their geometry by scanning electron microscopy (SEM) (Figure S1). The sparse substrate presents isolated wells separated by wide planar regions (Figure 1b and Figure S1a,b), allowing unambiguous spatial discrimination between curved and flat membrane domains. To extend this to proteome‐scale screening, the proportion of curved membrane area per substrate was increased by developing a dense patterned substrate featuring closely spaced hemispherical wells (Figure 1e). The dense substrate places wells in close proximity so that adjacent rims abut to form smooth, continuous ridges, thereby displaying positive, negative, and zero curvature simultaneously (Figure 1e and Figure S1c,d).

Supported lipid bilayers (SLBs) were formed on both substrates under identical conditions, and their quality was assessed using a fluorescently labeled lipid (Texas Red–DHPE) incorporated into each bilayer. Fluorescence recovery after photobleaching (FRAP) confirmed lateral membrane fluidity (Figure S2), yielding an apparent diffusion coefficient of 0.551 µm^2^/s. Although lower than values typically reported for planar SLBs (1–5 µm^2^/s), this difference is attributable to the larger actual membrane surface area relative to its 2D projection.

Bilayer continuity was confirmed via Texas Red–DHPE z‐stack imaging. 3D reconstruction (Figure S3 and Movie S1) revealed a continuous lipid bilayer spanning both ridges and well interiors with no detectable discontinuities. Because fluorescence intensity varies with membrane geometry owing to the axially elongated point spread function of the confocal microscope, protein localization was quantified as a protein‐to‐lipid enrichment factor by normalizing protein fluorescence to the co‐localized Texas Red–DHPE signal.

To benchmark the platform against well‐established curvature preferences, we examined two canonical BAR‐domain sensors. We first evaluated the spatial distribution of the I‐BAR domain of IRSp53, a negative‐curvature sensor [10, 24], on the sparse substrate by immunofluorescence and z‐stack confocal imaging (Figure 1c). Protein localization on topographical SLBs was quantified using a multimodal image‐analysis workflow that mapped the averaged protein‐to‐lipid enrichment factor onto the well surface geometry (Figure S4). The I‐BAR domain was enriched in the concave well interior (Figure 1d), consistent with its reported curvature preference. Conversely, the N‐BAR domain of amphiphysin—a positive‐curvature sensor [7]—was depleted from concave regions and enriched at positively curved well rims of the dense substrate (Figure 1f–g), recapitulating its curvature preference.

Because the positive curvature presented here (∼1.71 µm^−^^1^) is substantially lower than the preferred curvature of the N‐BAR domain (∼91 µm^−^^1^), the enrichment over flat regions was less sharply defined. Nevertheless, the expected localization pattern was still reproduced, indicating that the platform captures curvature preference even when the presented curvature does not fully match the optimal curvature of the protein. Together, these results validate a curvature‐resolved, area‐corrected readout for both positive‐ and negative‐curvature sensors. We next used this platform to screen proteins in cell lysates and identify curvature‐sensing proteins across the proteome without prior knowledge of the candidates.

### 2.3 Geometry‐Resolved Screening of Membrane Curvature Sensor Candidates from the Peripheral Membrane Proteins Fraction

To perform geometry‐resolved proteomic screening, we fabricated three substrates with distinct membrane geometry compositions (Figure 2a,b and Figure S1e–h). Substrate A (close‐packed) maximizes curved membrane area with minimal inter‐well spacing; Substrate B (semi‐dense) presents gentler curvature across wider wells; and Substrate C provides a flat membrane reference. Together, these substrates span predominantly curved, mixed, and exclusively flat membrane environments, enabling comparative proteomic analysis across the membrane curvature landscape.

Peripheral membrane proteins were extracted from the microsomal fraction of MDA‐MB‐231 cells to enrich membrane‐associated proteins while minimizing abundant soluble contaminants. The microsomal fraction comprises membranes derived primarily from the endoplasmic reticulum (ER), Golgi apparatus, and plasma membrane, while excluding many soluble complexes such as nuclear spliceosome proteins that have previously been reported as non‐specific binders in bead‐based curvature proteomics [19].

After filtering contaminants and low‐abundance proteins, a total of 671 unique proteins were identified across the three substrates. Among these, 396 (59%) were shared by all substrates, whereas 178 (27%) were detected exclusively on Substrate A (Figure 2c), consistent with its larger membrane area available for protein capture. Protein abundance was normalized to the total peptide amount to account for variations in overall protein recovery across substrates with distinct geometries. Pairwise comparison of the normalized abundances revealed curvature‐dependent protein enrichment (Figure 2d–f). Using thresholds of >1.5‐fold enrichment in either direction and adjusted *p*< 0.05, 71 non‐redundant candidate proteins were identified across the three substrate comparisons (41 from A/B, 48 from B/C, 30 from C/A). Importantly, these candidates spanned positive, negative, and zero curvature‐associated binding profiles, illustrating that the platform interrogates the full membrane‐curvature landscape rather than a single curvature sign.

### 2.4 Prioritization of Direct Curvature‐Sensing Candidates

Because differential enrichment alone cannot distinguish direct curvature sensors from indirectly recruited proteins, we established a prioritization workflow integrating hierarchical clustering, protein–protein interaction (PPI) networks, and protein annotation. As a first step, the 71 candidates were grouped by hierarchical clustering according to their binding profiles across the three substrates, yielding three major clusters, each showing a distinct preference for one of the three substrates—moderate curvature, flat, or high curvature (Figure 3a). These clusters also displayed characteristic subcellular localizations (Figure 3b,c), indicating that proteins sharing similar geometry preferences often originated from related membrane compartments.

Within each cluster, PPI networks were mapped using the STRING database (Figure 3d). Because highly connected proteins are more likely to represent secondary interactors within macromolecular complexes, disconnected or sparsely connected nodes were prioritized as candidate direct membrane geometry sensors. Although STRING connectivity is influenced by existing biological knowledge and therefore does not definitively distinguish direct from indirect interactions, this strategy provides a practical means of reducing false‐positive candidates.

This prioritization strategy was supported by two representative examples. First, the established curvature sensor EHD1 [25] was recovered as a disconnected node in Cluster 1, supporting the validity of the workflow. Because EHD1 has been extensively characterized as a membrane tubulation factor during endocytic recycling, it was not included in subsequent validation experiments. Second, SPTB2 (β‐II spectrin) appeared as a sparsely connected node in Cluster 2. Although SPTB2 is not typically classified as a curvature sensor, the non‐erythrocytic spectrin heterodimer formed with SPTA2 (α‐II spectrin) has previously been reported to preferentially bind large silica‐bead‐supported lipid bilayers (1000 nm) [26]. Because spectrin functions as an obligate α/β heterodimer, the SPTA2/SPTB2 complex was reconstituted for subsequent biochemical validation.

Candidate prioritization integrated four complementary criteria: hierarchical clustering, low intra‐cluster PPI connectivity (STRING), subcellular localization (UniProt), and domain architecture (SMART). Together, these filters selected seven representative candidates spanning distinct membrane geometry preferences, cellular localizations, and structural classes for biochemical validation (Table S1). Because biochemical validation required purified recombinant proteins, two candidates—mitochondrial ribosomal protein L45 (RM45) and endoplasmic reticulum resident protein 29 (ERP29)—could not be purified from HEK293T cells because of insufficient expression and were therefore excluded from subsequent analyses.

The remaining five were Rab‐14 (RAB14), microtubule‐associated protein RP/EB family member 1 (MARE1), annexin A1 (ANXA1), coronin‐1C (COR1C), and the non‐erythrocytic α‐II/β‐II spectrin heterodimer (SPTA2/SPTB2). The selected candidates predominantly localize to the plasma membrane and Golgi apparatus while representing diverse molecular functions. Importantly, none contains a canonical curvature‐sensing module such as a BAR domain or an ALPS motif, indicating that any geometry preference they display would arise from non‐canonical structural features. These candidates were subsequently examined using purified proteins to determine whether they intrinsically function as direct membrane geometry sensors.

### 2.5 Validation of the Prioritized Candidates

The proteomic screen was a single‐pass discovery step performed on cell lysates, in which binding was measured as bulk capture under competition among numerous cellular proteins. Furthermore, each substrate presented not a single uniform curvature, but rather a mixture of multiple curvature signs with varying magnitudes and surface areas. Consequently, enrichment on a given substrate cannot by itself be interpreted as a protein's intrinsic membrane geometry preference. To determine the intrinsic properties of the individual proteins, each prioritized candidate was expressed, purified, and assayed on the dense substrate that presents concave, convex, and planar regions on a single surface. Protein distribution was quantified from axial (XZ) confocal cross‐sections using curvature‐based segmentation of the surface profile (Figure 4a and Figures S5 and S6), and enrichment was expressed as log_2_(I_FITC_/I_TexasRed_). Enrichment was then compared across curvature classes using two‐sided Mann–Whitney U tests and Cohen's *d* values across at least 30 wells (Figure 4b–h).

Canonical BAR‐domain proteins were first assayed to calibrate the assay for both curvature signs. The I‐BAR domain was enriched in negatively curved regions relative to both flat and positively curved regions, as expected for a negative‐curvature sensor (Figure 4h). The N‐BAR domain showed the opposite polarity: it was significantly depleted from concave regions and localized to the positively curved pit rims, although its enrichment compared to flat regions did not reach statistical significance (*p* = 0.088; Figure 4g). These controls demonstrate that the assay reliably distinguishes positive from negative membrane curvature, while positive‐curvature enrichment is partially attenuated by the relatively gentle curvature presented on the substrate, reflecting a limitation in dynamic range rather than polarity.

Interestingly, among the prioritized candidates, four proteins—the SPTA2/SPTB2 spectrin heterodimer, ANXA1, COR1C, and MARE1—were significantly enriched on flat membranes relative to both positively and negatively curved regions, whereas RAB14 was selectively enriched on positively curved regions (Figure 4b–f). Conventional membrane‐curvature assays are inherently optimized to detect enrichment on highly curved membranes, making systematic identification of planar‐binding proteins difficult. The simultaneous identification of the positive curvature sensor RAB14 and the correct assignment of the canonical BAR domains demonstrate that these flat‐membrane preferences are not artifacts of the assay system. Notably, the planar preference of spectrin is consistent with our previous observations in a sedimentation assay using graded silica‐bead‐supported lipid bilayers [26]. Because that measurement used a different substrate geometry and a different detection principle, its agreement with the present results underscores the robustness of this finding and the reliability of this platform.

These results demonstrate that membrane curvature preference extends beyond positive‐ and negative‐curvature sensing to include proteins that preferentially recognize flat membrane regions. To visualize these relationships, the relative binding affinities across the three curvature regions were projected onto a ternary plot (Figure 4i). This mapping positions flat‐recognition—which we term “anti‐curvature” sensing—within the proteome not as a rare exception, but as a distinct class of curvature‐sensing proteins. Because the workflow simultaneously interrogates positive, negative, and zero curvature without candidate preselection, this class emerged directly from an unbiased discovery process. Therefore, this platform provides a geometry‐resolved workflow for the unbiased discovery and validation of membrane curvature sensors, enabling systematic investigation of protein–membrane interactions across different cell types, organelles, and lipid compositions.

## 3 Discussion

In this study, we established a geometry‐resolved supported lipid bilayer platform that advances research on membrane curvature sensing from targeted characterization to hypothesis‐agnostic, proteome‐scale discovery. By presenting convex, concave, and flat membrane geometries within a single continuous bilayer while providing sufficient curved membrane area for shotgun proteomics, the platform enables untargeted screening from complex cell‐derived protein fractions together with direct validation within the same geometric framework. Unlike previous approaches that focused on individual curvature classes or predefined candidates, this strategy systematically evaluates protein preference across the full membrane geometry landscape. To our knowledge, this is the first geometry‐resolved platform to integrate proteome‐scale discovery with biochemical validation of membrane geometry preference.

The power of this hypothesis‐free discovery strategy is illustrated by the identification of a previously overlooked class of membrane geometry sensors. Four of the five validated candidates preferentially associated with flat membranes rather than positively or negatively curved membranes. Such proteins are difficult to identify using conventional assays, which are inherently optimized to detect enrichment on highly curved membranes or evaluate only a single curvature sign. The molecular basis underlying these planar membrane preferences remains to be determined, and two non‐mutually exclusive scenarios can be considered: (i) active recognition, in which binding, self‐assembly, or oligomerization is promoted by planar geometry, and (ii) passive exclusion, in which proteins are sterically excluded from curved membranes. For example, ANXA1 assembles into 2D lattices on membranes [27, 28], COR1C forms trimers with extended planar footprints [29, 30, 31], and spectrin forms an extensive membrane‐associated cytoskeletal network [26]. These architectures suggest that oligomerization or higher‐order assembly may contribute to anti‐curvature sensing. Enhanced oligomerization on flat membranes could enlarge the protein footprint, thereby imposing steric hindrance on curved membranes and promoting enrichment on planar surfaces. The relative contributions of active recognition and passive exclusion could be assessed in future studies by perturbing oligomerization or higher‐order assembly and examining its effect on curvature‐dependent membrane binding. Together, these observations suggest that membrane geometry recognition extends beyond canonical curvature‐sensing domains and highlight the ability of unbiased proteome‐scale screening to uncover previously unrecognized modes of membrane geometry recognition.

RAB14 was identified as a positive‐curvature sensor, demonstrating that the platform can resolve multiple curvature preferences within a single experiment. Rab GTPases are not conventionally regarded as curvature sensors; however, their preference for positive curvature may be plausibly explained by prenylation‐dependent insertion into lipid‐packing defects on convex membranes, consistent with reports for RAB1, RAB5, and RAB6 [32]. This interpretation is consistent with the observation that C‐terminal tagging abolishes membrane binding (Figure S6). The identification of both anti‐curvature and positive‐curvature sensors demonstrates that the platform can resolve multiple and distinct modes of membrane geometry recognition within a single experimental framework.

Several limitations should be noted. Our screening was conducted using a single lipid composition and a single biological preparation; thus, proteins that require specific lipid environments may have remained undetected. The prioritization workflow was designed to favor sparsely connected proteins as candidate direct membrane curvature sensors, leaving unexplored the potential for cooperative curvature recognition. In addition, the dynamic range for detecting curvature preference of proteins is limited by the curvature magnitude of the substrate: due to constraints in lithographic fabrication and in the spatial resolution of the optical microscopy, the substrates were designed to present curvatures ranging from −1.48 to 1.71 µm^−^^1^ (Figure S1). As such, the current platform is more suitable for detecting proteins that preferentially sense moderate curvatures (e.g., on the order of a few µm^−^^1^) and is less sensitive to proteins preferring substantially higher curvatures (e.g., on the order of tens of µm^−^^1^).

In summary, our platform enables the direct capture of curvature‐sensing proteins from complex biological mixtures without requiring protein purification, thereby facilitating the identification of previously unrecognized membrane geometry sensors. An unbiased screen spanning the full range of membrane geometries—positive, negative, and zero curvature—allowed us to expand the concept of curvature sensing to encompass not only the conventional “accumulation on curved surfaces” but also a “preference for flat surfaces.” This expanded paradigm provides a broader conceptual framework for understanding how intracellular membrane geometry is recognized and organized. Looking forward, a systematic extension of this platform along two complementary axes—the proteome source and the lipid composition—provides a promising roadmap for future work. Screening protein fractions from different cell types or organelles against bilayers reconstituted with respective lipid compositions would illuminate how membrane geometry sensing varies across different cellular contexts. Furthermore, screening the same protein fraction against reconstituted membranes that mimic individual organelles and the plasma membrane would enable decoupling of the relative contributions of membrane curvature and lipid composition to protein–membrane interactions and function.

## 4 Methods

### 4.1 Recombinant Protein Expression and Purification from *E. coli*

The pGEX6P‐1 plasmid, encoding the N‐BAR domain of Amphiphysin, was previously reported [33]. Meanwhile, the pGEX6P‐1 plasmid encoding the I‐BAR domain of IRSp53 was gifted by Dr. Shiro Suetsugu (Nara Institute of Science and Technology).

For protein expression, these plasmids were transformed into *Escherichia coli* BL21 (DE3) cells (Nippon Gene, Tokyo, Japan, Cat. #318‐06531). The bacteria were cultured in Terrific Broth (TB) medium (Invitrogen, Thermo Fisher Scientific, Waltham, USA) containing 50 µg/mL ampicillin at 37°C. Once the optical density at 600 nm reached 0.5, protein expression was induced by 0.1 mM isopropyl β‐D‐1‐thiogalactopyranoside, followed by incubation overnight at 16°C. Cells were harvested by centrifugation at 8000 × *g* for 5 min. The cell pellet was resuspended in phosphate‐buffered saline (PBS) supplemented with ProteoGuard EDTA‐Free Protease Inhibitor Cocktail (Takara Bio, Kusatsu, Japan) and lysed by tip sonication. After removing cell debris through centrifugation at 20 000× *g* for 20 min, the supernatant was incubated with Glutathione Sepharose 4B beads (Cytiva, Tokyo, Japan) at 4°C overnight, with gentle rotation. Beads were washed twice with PBS, and the bound proteins were eluted in buffer (50 mM Tris‐HCl, pH 8.0, and 10 mM reduced glutathione) for 1 h at 4°C. Protein samples underwent dialysis with an 8 kDa Mini Dialysis Kit (Cytiva) to exchange the buffer for PBS. Finally, protein purity and identity were confirmed using 12% sodium dodecyl sulfate (SDS)–polyacrylamide gel electrophoresis (PAGE) and Coomassie Brilliant Blue (CBB) R‐250 staining (Bio‐Rad, Hercules, USA), respectively (Figure S7).

### 4.2 Mammalian Protein Expression and Purification from HEK293T Cells

Plasmids encoding *ANXA1* (RefSeq: NM_000700.3), *CORO1C* (RefSeq: NM_001105237.2), and *MAPRE1* (RefSeq: NM_012325.3), all cloned into pcDNA3.1‐C‐FLAG, as well as *RAB14* (RefSeq: NM_016322) cloned into both pcDNA3.1‐C‐FLAG and pcDNA3.1‐N‐FLAG, were obtained from GenScript (Piscataway, USA). The pCIG‐*SPTAN1*‐FLAG plasmid was kindly provided by Dr. Hirotomo Saitsu (Hamamatsu University School of Medicine, Hamamatsu, Japan). The *SPTBN1*‐HA plasmid was obtained from Addgene (Watertown, MA, USA; RRID: Addgene_31070). Human embryonic kidney 293T (HEK293T) cells (ECACC 12022001), tested negative for mycoplasma contamination and authenticated by the supplier, were obtained from the European Collection of Authenticated Cell Cultures (Salisbury, UK) through KAC Co., Ltd. (Kyoto, Japan).

To analyze protein expression, HEK293T cells were cultured in Dulbecco's modified Eagle medium (DMEM; Gibco) supplemented with 10% fetal bovine serum (FBS) and 100 U/mL penicillin–streptomycin (Fujifilm, Wako Pure Chemical, Osaka, Japan). HEK293T cells grown in 15 cm dishes were transfected using 12 µg plasmid DNA (encoding *ANXA1, CORO1C, MAPRE1, RAB14, SPTAN1*, or *SPTBN1*) and 180 µg PEI MAX (Polysciences, Warrington, USA). After 72 h at 37°C in 5% CO_2_ with daily media exchange, cells were harvested and lysed in Cell Lysis Buffer M (Wako) containing ProteoGuard EDTA‐Free Protease Inhibitor Cocktail (Takara Bio). For cells expressing N‐terminal FLAG‐tagged RAB14, 0.1% Triton X‐100 was added to the lysis buffer. Lysates were cleared by centrifugation at 20 000 × *g* for 30 min. Supernatants were then incubated with anti‐DYKDDDDK‐tag antibody beads (Wako) for 2 h at 4°C. Beads were washed three times with PBS and centrifuged at 8 000 × *g* for 1 min after each wash.

Given that spectrin senses membrane curvature as an SPTA2–SPTB2 heterodimer [26], the functional complex was reconstituted for in vitro validation. SPTA2‐FLAG was immobilized on anti‐DYKDDDDK beads and incubated with lysates containing SPTBN1‐HA for 2 h at 4°C. Bound proteins were eluted with 150 µg/mL DYKDDDDK peptide (Wako) for 1 h. Excess peptide was removed via dialysis with an 8 kDa Mini Dialysis Kit (Cytiva). Purified proteins were stored at 4°C and used within 7 days.

Protein purity and presence were confirmed by 12% SDS‐PAGE and Western blotting. The iBlot2 system (Thermo Fisher Scientific) and iBind Western Device (Thermo Fisher Scientific) facilitated blotting procedures. Primary antibodies included anti‐DYKDDDDK (1:2000; Proteintech Group, Rosemont, IL, USA, Cat. #66008‐4‐Ig) and anti‐HA (Cat. #66006‐2‐Ig). A peroxidase‐conjugated secondary antibody (Jackson ImmunoResearch, USA, Cat. #115‐035‐003) and Pierce 1‐Step Ultra TMB Blotting Solution (Thermo Fisher Scientific) were employed for signal detection (Figures S8 and S9).

### 4.3 Microsome Isolation from MDA‐MB‐231 Cells

MDA‐MB‐231 cells (ECACC 92020424; Sigma‐Aldrich, St. Louis, USA), tested negative for mycoplasma contamination and authenticated by the supplier, were cultured in DMEM (Gibco) supplemented with 10% FBS (Gibco) and 100 U/mL penicillin–streptomycin (Wako) at 37°C in a humidified atmosphere containing 5% CO_2_.

Cells grown in 15 cm dishes were harvested, washed with PBS, and processed using the Endoplasmic Reticulum Isolation Kit (Merck, Darmstadt, Germany, Cat. #ER0100) according to the manufacturer's instructions. Briefly, cells were resuspended in Hypotonic Extraction Buffer and incubated for 20 min at 4°C. Samples were then centrifuged at 600 × *g* for 5 min, and the resulting pellet was resuspended in Isotonic Extraction Buffer and homogenized with a Dounce homogenizer. The homogenate was centrifuged at 1000 × *g* for 10 min at 4°C to remove nuclei and intact cells. The supernatant was centrifuged at 12 000 × *g* for 15 min at 4°C, followed by ultracentrifugation at 100 000 × *g* for 60 min at 4°C. The final pellet was collected as a microsome fraction.

To isolate peripheral membrane proteins, the microsome pellet was resuspended in PBS and disrupted using a probe‐tip sonicator. Membranes were recovered by ultracentrifugation at 100 000 × *g* for 60 min at 4°C. To dissociate peripheral proteins, the pellet was resuspended in 5 mL of 2.5 M sodium borate and incubated for 2 h at 4°C. Following another ultracentrifugation step (100 000 × *g* for 60 min at 4°C), the supernatant containing detached proteins was collected. Buffer exchange with PBS was performed by overnight dialysis using an 8 kDa Mini Dialysis Kit (Cytiva).

### 4.4 Nanostructure Fabrication and Characterization

Four substrate variants were developed to accommodate distinct analytical workflows: a sparse‐patterned substrate with widely spaced wells for high‐resolution confocal characterization, a densely patterned substrate with closely spaced wells for fluorescence‐based evaluation of curvature sensing, and two proteomics substrates derived from the dense‐patterned design: Substrate A (close‐packed) featured minimized inter‐well spacing to substantially reduce flat regions, and Substrate B (semi‐dense) incorporated greater inter‐well separation and larger well diameters to produce lower curvature while retaining narrow features. An unpatterned, flat quartz substrate was designated as Substrate C. Detailed geometric characteristics and curvature profiles of all substrate variants are provided in Figure S1.

Patterned quartz substrates were fabricated using photolithography and reactive ion etching (RIE). Quartz substrates (15 mm × 15 mm × 0.17 mm) underwent ozone cleaning for 5 min and were coated with photoresist (OFPR‐8600LB; Tokyo Ohka Kogyo, Tokyo, Japan) via spin coating (800 rpm for 3 s followed by 7 000 rpm for 20 s). The coated substrates were pre‐baked at 95°C for 90 s. Hemispherical curvature patterns were defined through grayscale exposure using a maskless aligner (µMLA; Heidelberg Instruments, Heidelberg, Germany). After exposure, substrates were post‐baked at 115°C for 90 s and developed with 2.38% NMD‐3 (Tokyo Ohka Kogyo) for 90 s. Pattern transfer into the quartz substrate was accomplished by RIE (RIE‐10NR, Samco, Japan) under specified conditions: 4 Pa, 75 W, and gas flow rates of O_2_ (2 sccm), Ar (10 sccm), CHF_3_ (30 sccm), and CF_4_ (25 sccm) for 80 min. Substrate morphology and dimensions were characterized using SEM (GeminiSEM 460, Zeiss, Jena, Germany).

### 4.5 Preparation of Supported Lipid Bilayers (SLBs)

SLBs were prepared using the following lipid composition: 1,2‐dioleoyl‐sn‐glycero‐3‐phosphocholine (DOPC; Avanti Polar Lipids, Alabaster, USA, Cat. #850375P), 1,2‐dioleoyl‐sn‐glycero‐3‐phosphoethanolamine (DOPE; Tokyo Chemical Industry, Tokyo, Japan, Cat. #D4251), cholesterol (Avanti, Cat. #700000), and L‐α‐phosphatidylinositol‐4,5‐bisphosphate (PI(4,5)P_2_ Avanti, Cat. #840046), mixed at 40:40:10:10 (w/w%). Based on the average molecular weights of these lipids (786.1, 744.0, 386.7, and 1096.4 g/mol, respectively; PI(4,5)P_2_ supplied as the triammonium salt), this corresponds to approximately 36.4:38.5:18.5:6.5 mol%. This lipid composition was adopted from that used in previous studies characterizing membrane curvature‐dependent protein binding [7, 19]. For fluorescence imaging, Texas Red 1,2‐dihexadecanoyl‐sn‐glycero‐3‐phosphoethanolamine (Texas Red‐DHPE, Thermo Fisher Scientific) was added at a weight ratio of 1:100 (Texas Red‐DHPE: lipid mixture described above, ∼0.5 mol%). To prepare the lipid mixture, 0.4 mg of lipid was dried under an argon gas stream to form a lipid cake, which was stored at −30°C until use. To minimize the oxidation of PI(4,5)P_2_, aliquots of the lipid mixture were prepared from a newly opened vial purchased from the manufacturer, and SLBs were freshly prepared from the aliquots immediately before each experiment.

Before forming the SLBs, quartz substrates were sequentially cleaned by sonication in 2‐propanol for 20 min, followed by immersion in 1 M NaOH for 10 min and ∼30% H_2_O_2_ for 30 min. Substrates were rinsed with deionized water. The lipid cake was rehydrated in 800 µL PBS, and small unilamellar vesicles (SUVs) were formed using probe‐tip sonication on ice with protection from light. These SUVs were incubated on the cleaned quartz substrates at 50°C for 40 min, protected from light, enabling SLB formation via vesicle fusion. Excess vesicles were gently rinsed with PBS, ensuring membranes remained hydrated throughout the process.

### 4.6 Fluorescence Recovery After Photobleaching (FRAP)

Membrane fluidity was assessed by FRAP using an LSM780 confocal laser‐scanning microscope (Carl Zeiss) equipped with a 40× oil‐immersion objective lens (Alpha Plan‐Apochromat, NA 1.1). A circular region of interest (ROI, ∼10 µm in diameter) was defined for bleaching, together with a second ROI of the same size in an unbleached area that served as a reference. The bleach ROI was illuminated with a 561 nm laser at 100% power until its fluorescence intensity had decreased by 30%. From the resulting time‐lapse images, the mean fluorescence intensities of the bleach and reference ROIs were extracted at each time point, and the intensity of the bleach ROI was normalized to that of the reference ROI to correct for imaging‐induced photobleaching. The normalized recovery curve was then fitted with the following single‐exponential function to obtain the half‐recovery time (*t*_1/2_):

$$f\;\left(t\right)=f_{\infty }\;-\left(f_{\infty }-f_{0}\right)e^{-kt},\;\;t_{1/2}=\frac{\ln2}{k}$$

where *f*_∞_ is the fluorescence intensity at the recovery plateau and *f*_0_ is the minimum intensity immediately after bleaching. The apparent diffusion coefficient (*D*) was calculated using the following equation reported previously [34]:

$$D=0.224w^{2}t_{1/2}$$

where *w* is the radius of the bleach ROI.

### 4.7 SLB Recruitment and Elution of Peripheral Membrane Proteins

The proteomics experiment was designed as a single‐pass discovery screen to identify candidate curvature‐sensing proteins for subsequent independent validation. One biological preparation was used, with three technical replicates per substrate geometry to ensure analytical reproducibility. For protein screening, 150 µL of the microsome peripheral membrane protein fraction (∼15 µg of protein) was incubated on SLBs formed on the three proteomics substrates (A, B, and C) at 37°C for 1 h in the dark. After incubation, unbound proteins were removed by gently rinsing the substrates twice with PBS. Bound proteins were eluted by adding 150 µL of 1% SDS to the substrate and heating the mixture at 95°C for 5 min. The resulting eluate was collected, precipitated by mixing with 1.4 mL of ice‐cold acetone, and stored at −80°C for 90 min. Protein precipitates were recovered by centrifugation at 18 000 × *g* for 30 min at 4°C. The final pellet was resuspended in 46 µL of 1% SDS for further analysis.

### 4.8 Proteomic Sample Preparation and LC‐MS/MS Analysis

Sample preparation was conducted following the SP3 method [35]. Protein samples were reduced with tris(2‐carboxyethyl)phosphine (TCEP) at a final concentration of 2.5 mM and incubated at 37°C for 30 min. Alkylation was then performed by adding 1 µL of 1 M iodoacetamide and incubating in the dark at 24°C for 30 min. For protein purification, 2 µL of Sera‐Mag SpeedBead Carboxylate‐Modified Magnetic Particles (Cytiva) and ethanol were added to a final volume of 100 µL. The mixture was agitated at 1000 rpm for 5 min at 24°C. The supernatant was discarded, and the beads were washed three times with 180 µL of 80% ethanol. The washed beads were resuspended in 100 µL of 50 mM ammonium bicarbonate buffer containing 0.8 µg of Trypsin/Lys‐C Mix (Promega, Madison, USA, Cat. #V5072) and incubated overnight at 37°C. After digestion, peptides were collected by centrifugation at 15 000 rpm for 1 min with magnetic separation, and the reaction was quenched with 5 µL of 10% trifluoroacetic acid (TFA). Peptides were desalted using a GL‐Tip SDB (GL Sciences, Tokyo, Japan) that had been pre‐activated with 80% acetonitrile (ACN) containing 0.1% TFA and equilibrated with 2% ACN containing 0.1% TFA. After sample loading, the tip was washed twice with the equilibration solution, and peptides were eluted with 80% ACN containing 0.1% TFA. The eluate was dried using a SpeedVac and reconstituted in 15 µL of 2% ACN containing 0.1% TFA. After centrifugation at 20 000 × g for 5 min at 4°C to remove insoluble material, the supernatant was subjected to LC‐MS/MS analysis.

LC‐MS/MS measurement was performed using a Q‐Exactive tandem mass spectrometer and an Easy‐nLC 1000 nano‐HPLC system (Thermo Fisher Scientific). The trap column for nano‐HPLC was a 2 cm × 75 µm capillary column packed with 3 µm C18 silica particles (Thermo Fisher Scientific), and the separation column was a 12.5 cm × 75 µm capillary column packed with 3 µm C18 silica particles (Nikkyo Technos, Tokyo, Japan). The flow rate for the nano‐HPLC was 300 nL/min. Separation was performed using a linear acetonitrile gradient ranging from 10% to 40% over 30 min in the presence of 0.1% formic acid. Nano‐LC‐MS/MS data were acquired in data‐dependent acquisition (DDA) mode controlled by the Xcalibur 4.0 software (Thermo Fisher Scientific). The DDA settings were as follows: the resolution was 70 000 for full MS scans and 17,500 for MS2 scans. The AGC target was 3.0E6 for full MS scans and 5.0E5 for MS2 scans. The maximum IT was 60 ms for both full MS scans and MS2 scans. The range for full MS scans was 310–1500 m/z. The top 10 signals from each full MS scan were selected for MS2 scans. Dynamic exclusion was set to 10 s. Only +2 to +4 charge ions were included for MS2 measurement. DDA measurements were performed three times for each sample. Mass spectrometry data were deposited in the Japan Proteome Standard Repository and Database (jPOST) [36].

### 4.9 Prioritization of Candidate Proteins

Protein identification and label‐free quantification (LFQ) were performed using Proteome Discoverer (version 3.1; Thermo Fisher) bundled with the Sequest HT search engine. The amino acid sequence database for the peptide search was obtained from the UniProt database (https://www.uniprot.org/, downloaded on December 28, 2023). To mitigate technical variability in sample loading and instrument sensitivity, protein abundances were normalized using the “Total Peptide Amount” method integrated within the software. The resulting normalized abundance values, reflecting total signal‐normalized intensities, served as the basis for subsequent statistical analyses.

Proteins were filtered to ensure high‐confidence identification; specifically, only those with at least six peptide‐spectrum matches (PSMs) and two or more unique peptides were retained. Common laboratory contaminants, such as keratins, were systematically excluded from further analysis.

To identify differentially abundant proteins among the three proteomic substrates (Substrates A, B, and C), normalized abundance data were compared using two‐sided Student's *t*‐tests. Multiple testing corrections were applied through the Benjamini–Hochberg procedure to control the false discovery rate (FDR). Proteins with an absolute fold change greater than 1.5 and an adjusted *p*< 0.05 were considered significantly enriched and visualized in volcano plots.

Significantly enriched proteins underwent hierarchical clustering based on Z‐scores of normalized abundances, using Euclidean distance and Ward's linkage. To avoid arbitrary threshold selection and to ensure robustness against differences in cluster size, the dendrogram was traversed from the root, and each branch was cut at the merge showing the largest drop in fusion distance (Figure S10). Subcellular localization was annotated using the UniProt database (https://www.uniprot.org/), while PPI networks were constructed and visualized using the STRING database (version 12.0; https://string‐db.org/). Protein domains were obtained from the SMART database (https://smart.embl.de/). All statistical analyses and data visualizations were performed using Python (version 3.11) with relevant libraries including NumPy, SciPy, Pandas, Matplotlib, Seaborn, and StatsModels.

### 4.10 In Vitro Curvature‐Sensing Assays With Purified Proteins

All incubation and immunostaining steps were carried out in the dark to minimize photobleaching of fluorescent probes and photooxidation of PI(4,5)P_2_. Purified protein solutions (50 µg/mL) were incubated on SLBs formed on the dense‐patterned substrates at 37°C for 20 min. Excess proteins were removed by rinsing the substrate twice with PBS. The substrates were incubated with primary antibodies against DYKDDDDK (1:1000; Proteintech, Cat. #66008‐4‐Ig) or GST (1:1000; Proteintech, Cat. #66001‐2‐Ig), diluted in PBS, for 20 min at 37°C. After washing twice with PBS, a fluorescently labeled secondary antibody (anti‐mouse IgG, 1:500; Proteintech, Cat. #SA00003‐1) was applied and incubated for 20 min at 37°C. After three thorough PBS washes, the substrates were covered with VECTASHIELD Mounting Medium (Vector Laboratories, Newark, USA, Cat. #H‐1700) and mounted on glass slides for imaging.

### 4.11 Confocal Microscopy and Image Quantification

Fluorescence images were acquired using an LSM780 confocal laser‐scanning microscope equipped with an Airyscan detector (Zeiss). Imaging was conducted utilizing a 100× oil‐immersion objective lens (Alpha Plan‐Apochromat, NA1.46). Z‐stack images for 3D reconstruction were collected with a vertical interval of 0.5 µm. FITC (protein) and Texas Red (lipid) channels were excited using 488 and 561 nm lasers, respectively. To improve the signal‐to‐noise ratio, the Airyscan unit was operated in super‐resolution mode.

Image processing and X–Z plane reconstruction were performed using custom Python scripts (version 3.11), employing libraries such as NumPy, OpenCV, SciPy, statsmodels, scikit‐image, and Matplotlib. Maximum‐intensity projections (MIPs) and X–Z cross‐sectional views were generated to visualize protein recruitment to curved membranes. To analyze curvature‐dependent protein enrichment, surface geometry masks were generated from SEM images of the corresponding substrate variant (sparse‐patterned for I‐BAR; dense‐patterned for N‐BAR) and of candidate proteins. These masks were spatially aligned with reconstructed X–Z fluorescence images. Fluorescence intensities from FITC and Texas Red channels were averaged across 30 independent wells and normalized within each X–Z image. The protein enrichment factor was defined as the log_2_ ratio of normalized FITC intensity to Texas Red (lipid) intensity (log_2_[I_FITC_/I_TexasRed_]).

Local surface curvatures were calculated directly from the geometry mask. Log_2_ enrichment factors were classified into three categories based on local curvature: flat, positively curved, and negatively curved. Statistical significance was determined using the two‐sided Mann–Whitney U test with Bonferroni correction for multiple comparisons.

## Author Contributions

**Takumi Komikawa**: Conceptualization, Formal analysis, Investigation, Methodology, Writing – original draft. **Rikuto Kawakami**: Investigation, Resources. **Kunanon Chattrairat**: Investigation, Resources. **Tatsuya Niwa**: Formal analysis, Methodology, Software. Writing – review & editing. **Taiga Ajiri**: Methodology, Resources. **Takao Yasui**: Funding Acquisition, Supervision, Writing – review & editing. **Masayoshi Tanaka**: Conceptualization, Funding Acquisition, Supervision, Writing – review & editing

## Conflicts of Interest

The authors declare no conflicts of interest.

## Supporting information




**Supporting File 1**: advs77654‐sup‐0001‐SuppMat.docx.

**Supporting File 2**: advs77654‐sup‐0002‐Movie.avi.

## Data Availability

The mass spectrometry proteomics data have been deposited in the Japan Proteome Standard Repository and Database (jPOST) under accession number JPST004605 (PXD078093 for ProteomeXchange). All custom Python scripts used in this study are deposited in Zenodo at https://doi.org/10.5281/zenodo.20023875.
