# Supplementary material for: An Unbiased Geometry‐Resolved Membrane Platform for Proteome‐Scale Discovery of Membrane Curvature Sensors
Source: Adv Sci (Weinh). 2026 Sep 8:e77654. Online ahead of print. doi: 10.1002/advs.77654 (PMC13554497; doi:10.1002/advs.77654)
Supplement: Supplementary file 1 [file ADVS-9999-e77654-s002.docx]

**Supplementary Information**

**An Unbiased Geometry-Resolved Membrane Platform for Proteome-Scale Discovery of Membrane Curvature Sensors**

Takumi Komikawa^1^, Rikuto Kawakami^1^, Kunanon Chattrairat^2^, Tatsuya Niwa^3^, Taiga Ajiri^2^, Takao Yasui^2^, Masayoshi Tanaka^1,*^

^1^School of Materials and Chemical Technology, Institute of Science Tokyo, 4259 Nagatsuta, Midori-ku, Yokohama 226-8503, Japan

^2^Graduate School of Engineering, Nagoya University, Furo, Chikusa, Nagoya, 464-8603, Japan

^3^Institute of Integrated Research, Institute of Science Tokyo, 4259 Nagatsuta, Midori-ku, Yokohama 226-8503, Japan

*Corresponding author: [tanaka.m@mct.isct.ac.jp](mailto:tanaka.m@mct.isct.ac.jp)

**Supplementary Note | Estimation of Required SLB Surface Area for Proteomics Analysis**

To estimate the required supported lipid bilayer (SLB) surface area for proteomics analysis, we set several assumptions. It should be noted that protein binding to SLBs does not always result in close packing but rather random adsorption; at the same time, protein binding may not be limited to a single layer. This calculation aims to determine the order of magnitude of the required area; thus, close packing in a single layer is assumed as a first approximation.

**Prerequisites:**

- Minimum protein amount required for LC-MS/MS analysis: 0.5 µg
- Average molecular weight (Mw) of proteins: 50 kDa
- Approximate diameter of a 50 kDa globular protein: 5 nm [1]

**Calculation:**

The number of molecules in 0.5 µg of protein with an average Mw of 50 kDa is:

$$N=\frac{0.5\times{10}^{-6}}{50,000}\times6.02\times{10}^{23}\approx6.0\times{10}^{12} \mathrm{molecules}$$

Assuming close packing on a planar surface, the area occupied per molecule is approximately:

$$A_{\mathrm{molecules}}=\left( 5\times{10}^{-6}\mathrm{mm} \right)^{2}=2.5\times{10}^{-11} mm^{2}$$

The total surface area required is therefore:

$$A_{\mathrm{total}}=N\times A_{\mathrm{molecule}}\approx150 mm^{2}$$

This estimate indicates that an SLB surface area on the order of **150 mm²** is required to capture 0.5 µg of protein under the close-packing assumption. In practice, because random adsorption results in lower surface coverage than ideal close packing, the actual required area may be larger. Conversely, multilayer binding could partially compensate for this effect. Nevertheless, these factors are expected to remain within the same order of magnitude, and thus an SLB surface area of approximately 150 mm² provides a reasonable target for the experimental design.

**Supplementary Figures**

**Figure S1:** **Curvature characterization of nanopatterned quartz substrates**. **a, c, e, g**, Representative SEM images of the sparse substrate (a), dense substrate (c), Proteomic Substrate A (close-packed; e) and Proteomic Substrate B (semi-dense; g). For each substrate, top views (left) and tilted cross-sectional views (right) are shown. Green lines indicate the surface profiles extracted from cross-sectional images for curvature calculation. Scale bars are shown in each panel. **b, d, f, h**, Fitted curvature profiles derived from the extracted surface geometries (green lines) for the sparse substrate (b), dense substrate (d), Proteomic Substrate A (f) and Proteomic Substrate B (h). Negative curvature (purple) and positive curvature (green) regions are indicated, with κ values (µm⁻¹) annotated. Flat regions (κ = 0) are shown in grey where applicable.

**Figure S2: Fluorescence recovery after photobleaching (FRAP) analysis of a lipid bilayer containing Texas Red-DHPE on a sparse substrate. a**, Representative cropped confocal images acquired before bleaching (i), immediately after bleaching (ii), and 15 s after bleaching (iii). The white circle indicates the bleached region of interest (ROI); the reference ROI, set outside this field of view, is not shown. **b**. Fluorescence intensity profiles of the bleached (orange) and reference (gray) ROIs over time. The gray-shaded region indicates the duration of bleaching. **c**, Exponential fit of the normalized fluorescence recovery curve for the bleached ROI.

**Figure S3: Serial *z*-slice images of the supported lipid bilayer on the dense-patterned substrate.** Individual optical sections (*z* = 1–9; 0.5 µm intervals) from the Texas Red–DHPE fluorescence *z*-stack acquired on the dense substrate (Supplementary Fig. S1c). Slice numbering proceeds from the substrate side (*z* = 1) toward the objective (*z* = 9). The three-dimensional reconstruction of this stack is presented in Movie S1. Scale bar, 2 µm.

**Figure S4: Schematic workflow of the multimodal image analysis for protein curvature sensing on topological SLBs.** X-Z fluorescence images generated from z-stacks were masked using the surface topography extracted from cross-sectional SEM images. The fluorescence intensity of each channel was normalized, and the protein (FITC) to lipid (Texas Red) ratio was calculated. These ratios were averaged over 30 wells and mapped onto the well surface geometry as log2(I_FITC_ / I_TexasRed_).

**Figure S5: Fluorescence imaging of candidate curvature-sensing proteins on patterned SLBs. a–d,** Representative confocal fluorescence images of ANXA1-FLAG (a), COR1C-FLAG (b), MARE1-FLAG (c), and the SPTA2-FLAG/SPTB2-HA heterodimer (d) on the dense-patterned substrate. Top: XZ orthogonal views; bottom: maximum intensity projections (MIP). Red and green signals indicate lipids (Texas Red–DHPE) and proteins (FITC-conjugated secondary antibody), respectively. Scale bar, 2 µm. Fluorescence images of RAB14 are presented in Supplementary Fig. 4.

**Figure S6: C-terminal prenylation is essential for the curvature-sensitive membrane recruitment of RAB14**. **a, b**, Representative fluorescence images of N-terminally tagged (FLAG-RAB14; a) and C-terminally tagged (RAB14-FLAG; b) proteins on patterned SLBs. Top: XZ orthogonal views; bottom: maximum intensity projections (MIP). Red and green signals indicate lipids (Texas Red) and proteins (FITC), respectively. Arrows indicate the positions of the XZ cross-sections. Scale bar, 2 µm. To allow direct comparison of binding affinities, the FITC and Texas Red channels in both a and b are displayed using identical brightness and contrast settings. For visualization of the weak RAB14-FLAG signal, a "Contrast enhanced" panel is provided; however, the extreme contrast enhancement required to resolve this signal results in minor spectral bleed-through from the Texas Red channel into the FITC channel. **c,** Schematic model illustrating the interaction between RAB14 variants and the lipid bilayer. The C-terminal prenyl (geranylgeranyl) group (magenta) is present in FLAG-RAB14, whereas C-terminal tagging (RAB14-FLAG) sterically hinders post-translational modification, leading to impaired membrane anchoring and a subsequent reduction in curvature sensitivity.

**Figure. S7: Purification of GST-tagged BAR-domain proteins. a,b,** SDS-PAGE analysis of purified GST–I-BAR (IRSp53) (a) and GST–N-BAR (Amphiphysin) (b) stained with Coomassie Brilliant Blue R-250. Lanes from left to right: molecular weight marker, insoluble fraction, soluble fraction, elution. Arrowheads indicate the target proteins (~53 kDa and ~54 kDa, respectively). Molecular weight markers (kDa) are shown on the left.

**Figure S8: Purification of FLAG-tagged candidate proteins. a–e,** SDS-PAGE analysis (left, Coomassie Brilliant Blue R-250 staining) and anti-FLAG western blot of the corresponding lysate (right) for ANXA1-FLAG (a), COR1C-FLAG (b), MARE1-FLAG (c), RAB14-FLAG (d), and FLAG-RAB14 (e). Arrowheads indicate the target proteins (ANXA1, ~39 kDa; COR1C, ~53 kDa; MARE1, ~30 kDa; RAB14, ~24 kDa). Lanes from left to right: lysate (1), flow-through (2), beads (3), and elution (4). Non-contiguous lanes from the same gel or membrane are separated by white lines. Molecular weight markers (kDa) are shown on the left.

**Figure S9: *In vitro* reconstitution of the SPTA2/SPTB2 spectrin heterodimer. a,** Schematic illustration of the sequential affinity purification strategy. SPTA2-FLAG was first captured on anti-DYKDDDDK beads, followed by incubation with SPTB2-HA lysate to reconstitute the heterodimer on-bead. The complex was recovered by competitive elution with DYKDDDDK peptide. **b,c**, SDS-PAGE analysis (b, Coomassie Brilliant Blue R-250 staining), anti-FLAG western blot (c, left), and anti-HA western blot (c, right). Lanes: SPTA2-FLAG single elution (1), SPTB2-HA lysate (2), and SPTA2/SPTB co-elution (3). Co-detection of both SPTA2 and SPTB2 in lane 3 by anti-FLAG and anti-HA blotting confirms successful heterodimer reconstitution. Arrowheads indicate SPTA2 (~283 kDa) and SPTB2 (~273 kDa). Non-contiguous lanes from the same gel or membrane are separated by white lines. Molecular weight markers (kDa) are shown on the left.

**Figure S10: Dendrogram of hierarchical clustering based on Ward's method with Euclidean distances.** Arrowheads indicate the largest gap in fusion (merging) distance within each branch.

**Movie S1: Three-dimensional reconstruction of the supported lipid bilayer on the dense-patterned substrate.** Texas Red–DHPE fluorescence *z*-stack images acquired on the dense substrate (Fig. S1c) were processed and rendered as a rotating 3D surface using ImageJ/FIJI. Each *z*-slice was denoised with a Gaussian filter and the brightness and contrast were uniformly adjusted before reconstruction using the 3D Viewer plugin in surface rendering mode. Individual *z*-slice images are presented in Fig. S3.

**Table S1: Prioritized curvature sensor candidates.**

*Proteins in gray-hatched rows were excluded from subsequent analysis due to insufficient yield from HEK293T expression and purification.

**Supplementary References**

[1] Erickson, H. P. Size and shape of protein molecules at the nanometer level determined by sedimentation, gel filtration, and electron microscopy. *Biol. Proced. Online* **11**, 32–51 (2009)
